# Cut and paste: A novel method of re-attaching rectus muscles with cyanoacrylate during recessions in strabismus

**DOI:** 10.4103/0301-4738.67051

**Published:** 2010

**Authors:** Anjum Darakshan, Abadan K Amitava

**Affiliations:** Institute of Ophthalmology, JN Medical College, Aligarh Muslim University, Aligarh, UP, India

**Keywords:** Bioadhesives, cyanoacrylate, polyglactin, recession, strabismus

## Abstract

**Aim:**

Bio-adhesives like cyanoacrylate offer an alternative to sutures to attach tissues, including in ophthalmology. This prospective trial evaluated the suitability and bio-tolerance of iso-amyl cyanoacrylate in rectus muscle recession surgery for strabismus.

**Materials and Methods:**

We randomized one eye in each of 10 cases of bilateral horizontal rectus recessions to 6/0 polyglactin and the other to iso-amyl-cyanoacrylate. We compared time to reattachment (from disinsertion), complications and inflammatory scores (0 to +3: nil, mild, moderate and severe) on Day One, at two and at four to six weeks post surgery.

**Results:**

There were no significant group differences in inflammatory scores (Wilcoxon, all values of *P*>0.05). All attachments held firm. Gluing took significantly longer by 5.24±1.91 min (95% CI for difference: 3.87-6.61). There were no complications.

**Conclusion:**

We feel that although it takes marginally longer, iso-amyl cyanoacrylate offers an effective and safe alternative to sutures for muscle recession in strabismus surgery. Since it is cheaper (vs. polyglactin) and offers multi-use possibility it may also prove to be cost-effective.

Strabismus surgery has evolved from suture-less myotomies and tenotomies to suture-needing (usually polyglactin) advancement, pleating and resections.[1] It appears that the wheel is set to turn full circle with a return to a suture-less option, by utilizing bio-adhesives to achieve scleral muscle attachments.[2] This may bring down both costs and needle-associated complications.

Despite a plethora of bio-adhesives,[3–10] we chose cyanoacrylate since it has been extensively used in ophthalmic surgery.[11–14] Moreover, it has anti-bacterial properties[15,16] and provides flexible bonding.[17]

Before the study we assessed the strength of the bond formed between the muscle and sclera on goats’ eyes using iso-amyl cyanoacrylate (IAC) and found it could withstand loads of up to 200 g, far more than the maximum force of 75 g generated by the human extra-ocular muscles.[18]

We designed a paired study to compare the suitability and bio-tolerance of using IAC (Novocryl; Alkem, Mumbai) compared to polyglactin sutures (PGS) (6-0 Vicryl, NW 2670; Johnson and Johnson, Aurangabad) in achieving muscle-sclera re-attachment in strabismus surgery.

## Materials and Methods

After ethical approval from the institutional review committee, and obtaining informed consent, we recruited 10 patients qualifying for bilateral symmetric horizontal muscle recession surgery. We excluded patients unable to cooperate, and those with evidence of systemic diseases, ocular inflammatory conditions, past surgeries, hormonal imbalances, or allergic diathesis. One eye was randomized (with a non-biased coin) to undergo re-attachment by sutures, while the other automatically qualified for the adhesive.

A detailed history and examination was carried out, including visual acuity (VA), refraction, biomicroscopy and ophthalmoscopy. Strabismus-specific information such as amount of strabismus, status of binocularity (with Bagolini glasses) and any treatment undertaken in the form of spectacles, prism or patching was recorded.

**Figure 1:**
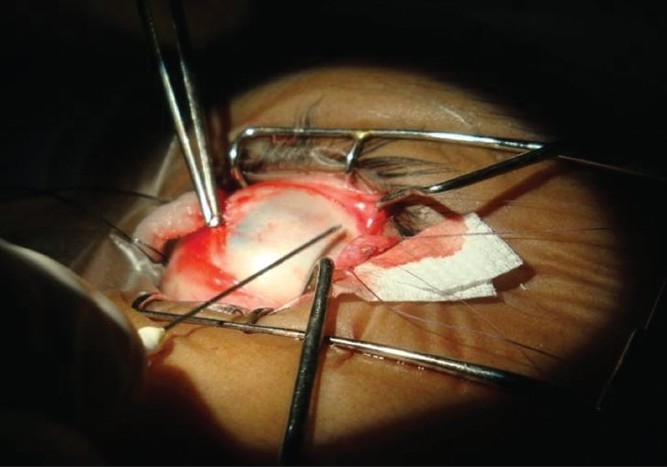
Iso-amyl cyanoacrylate being applied to the edge of the disinserted muscle edge with a 25G needle

**Figure 2:**
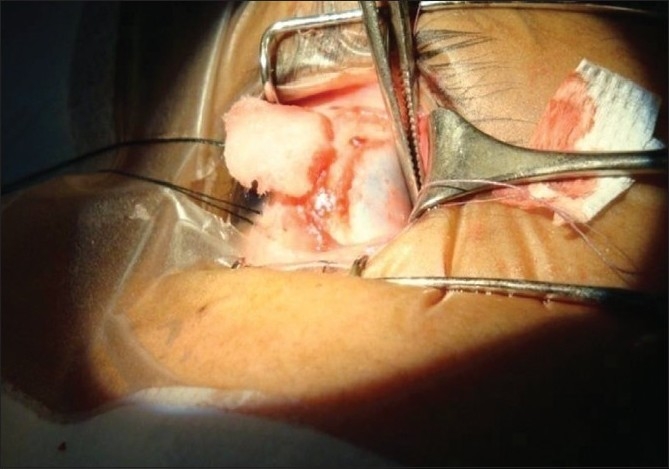
Pressure being applied on the muscle-sclera site after application of iso-amyl cyanoacrylate

Surgery was performed under standard peribulbar block. Using 5-0 silk traction sutures at 12 and 6 o’clock, the muscle was approached by the limbal approach. Prior to disinsertion, we passed a 6-0 double-armed Vicryl suture as follows: first a bite through the central belly was taken and then a whip stitch passed on the two edges of the tendon, near the insertion. We noted the time at disinsertion in order to calculate the length of time for reattachment of the muscle. For the study (IAC) group, we dried both the muscle and the sclera with cellulose strips and marked the point of required recession on the sclera after measuring with calipers. After applying one drop of IAC to both the scleral site and the cut edge of the muscle with a 25-gauge cannula [Fig. 1], we held them in apposition for 45 sec [Fig. 2]. In case there appeared to be a good ‘take’ evident from a good adhesion, no peeling-off appearance of the edge of the muscle, and a global movement on gently tugging at the (pre-placed) 6-0 Vicryl suture, then time to completion of adhesion was noted (glue time: GT). In the event that the attachment failed, we planned to re-apply once more before switching to standard sutural recession surgery. The 6-0 Vicryl sutures were passed through the insertion stump as a backup option to be used as an adjustable hang-back suture, in the unlikely event of the bioadhesive giving way. The ends were left long and carefully attached (with micropore) to the forehead at the end of surgery, and were to be removed after 4-6 h, if not needed. In the eye constituting the control (PGS) group, we sutured the disinserted muscle to the sclera with the 6-0 Vicryl in a standard cross sword fashion. At the end, time to completion of suturing was noted (Vicryl time: VT). We reattached the conjunctiva at two points with Vicryl (8-0) suture. The eyes were patched for 4 to 6 h. A combination of ofloxacin (Oflacin, Microvision, India) with ketorolac (Ketoflox: Allergan, India) drops were applied four times a day for at least 15 days.

Outcomes assessed included conjunctival injection, chemosis, discomfort and discharge, on a scale of 0 to 3 (none, mild, moderate and severe) on Day one, at two weeks, and at four to six weeks postoperatively. In addition the sum of individual scores constituted a ‘total score’ (TS) with a range 0 to 12. Intra-operatively we recorded GT and VT, as also complications if any.

We applied the Wilcoxon test and paired t-test for analyses. Significance was set at *P* ≤ 0.05. Confidence intervals (95% CI) are quoted where appropriate.

## Result

The demographic and clinical details of the 10 patients are outlined in Table 1. There were equal number of esotropes and exotropes. The median age was 18.5 years (range: 12-25).

**Table 1:** Baseline demographic and clinical characteristics of the 10 patients

Age/gender	BCVA* (with appropriate correction where prescribed)		Diagnosis	Bilateral Recession Plan
	RE	LE		
21y/M	Plano 20/20	Plano 20/20	XT† 45^∆^	LR§8 mm
18y/F	Plano 20/20	Plano 20/20	ET‡ 35^∆^	MR║5 mm
18y/m	-3DS 20/20	-3DS 20/20	XT 55^∆^	LR 10 mm
22y /F	+0.5DS,+0.5DC ×180° 20/20	+1.5DC×180° 20/20	XT 50^∆ 9 mm^	LR
20y /F	-15DS -2.5DC× 90° 20/40	-61.0DC×140° 20/60	XT 45^∆^	LR 8 mm
15y/F	+2 DS 20/30	+3DS+2DC ×120° 20/80	ET 55^∆^	MR 6.5 mm
12y/F	Plano 20/20	Plano 20/20	XT 35^∆^	LR 7.5 mm
20y/M	Plano 20/20	Plano 20/20	ET 50 ∆	MR 6 mm
18y/M	Plano 20/20	Plano 20/20	ET 35^∆^	MR 5 mm
19y/M	Plano 20/20	Plano 20/20	XT 45^∆^	LR 8 mm

* BCVA: Best corrected visual acuity

† XT: exotropia

‡ ET: esotropia

§ LR: Lateral rectus

║ MR: Medial rectus.

Analyses of individual inflammatory scores revealed no significant difference between the IAC and PGS groups at any point of follow-up. This is borne out by the Wilcoxon paired analyses. *P* values for injection, chemosis, discomfort and discharge (in the order stated) were all >0.05: on Day one: 0.10, 008, 0.32, 1.0; at Week two: 1.0, 0.32, 0.32, 1.0; and at Weeks 4-6: 0.32, 1.0, 1.0, 1.0.

**Figure 3:**
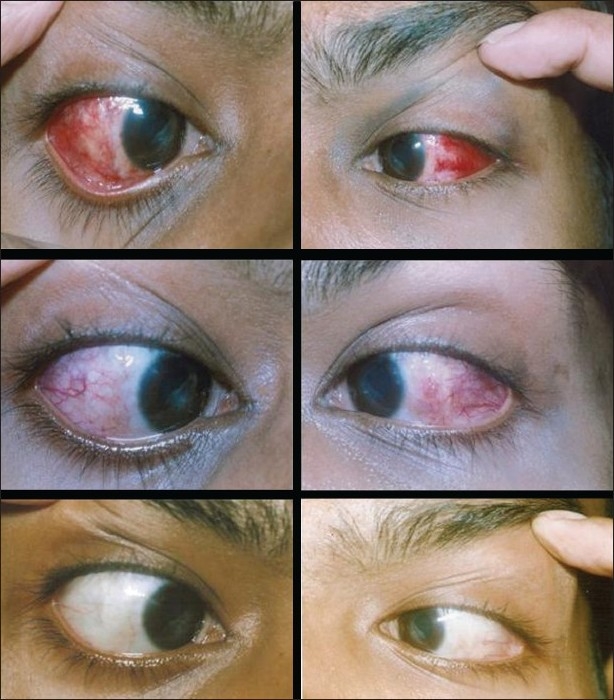
Postoperative follow-up of the same patient: RE: sutured, LE: glued. Top row — first day; middle row, at two weeks and bottom row at 4-6 weeks

On analyzing TS on the first postoperative day a significant difference (*P*=0.011) was evident favoring the glued eye. The mean TS for glued eyes was 4.4 (±1.17) while for PGS it was 5.2 (±0.79), with a mean of difference 0.80 (±0.63) (95%CI for difference: 0.35-1.25). Similar analyses of TS yielded no significant differences at two weeks (95%CI: -0.25 to 0.65) and at four to six weeks (95%CI: -0.33 to 0.13). The appearance of both the eyes of one patient during follow-up is shown in Fig. 3.

The mean VT of 5.04 (±1.70) min (range: 2.7 to 7.94), was significantly shorter than the mean GT of 10.29 (±2.59) min (range: 6 to 15): The 95% CI for paired difference was 3.87 to 6.61. None of the patients had any complication.

## Discussions

We could access only one comparable human study in the literature done by Mulet.[19] In contrast to us, they used the cyanoacrylate, Adal-1 (a mix of ethylcarboxyacrylate + ethylcyanoacrylate). Unlike ours, theirs was an unpaired study on 27 eyes: 17 sutured versus 10 glued recession surgeries.

Animal studies have yielded evidence both against,[18] and in support of,[20,21] an effective bond between rectus muscles and sclera using cyanoacrylate. Although not part of the protocol, we are aware that even after six months, the glued eyes demonstrate equally good ductions and versions comparable to the sutured eyes, suggesting effective bonding.

We could not demonstrate any difference in individual inflammatory scores between sutured eyes and glued eyes. Only the TS on the first day marginally favored the IAC group, and although statistically significant, the result was too small to be clinically meaningful. Comparative bio-tolerance has also been demonstrated in rabbits and humans, with statistically no significant difference in postoperative inflammatory reaction between cyanoacrylate and sutures.[4,19]

Though we did not measure the muscular displacement from the points of attachment, both Mulet (in humans) and Tolleni (in rabbits) failed to demonstrate any significant displacement after gluing.[20,21]

Compared to suturing, on an average, it took statistically significantly longer to glue back the muscles. We feel that a difference of a mere five minutes on an average would not be of much clinical importance. We are of the opinion that diligent cauterization of bleeders, (cautery was not part of our protocol) is likely to markedly decrease the total time for attachment with glue. We could not find any study where duration of surgery had been studied.

Although we did not undertake formal cost-effectiveness analysis, we estimate that use of IAC is likely to be cheaper than the use of Vicryl sutures: One 0.25 ml vial of IAC costs Rs. 250, and can suffice for both the eyes compared to Rs. 940 for the cost of two Vicryl sutures for two eyes. Clustering the patients on a particular day may allow greater cost reduction, since one IAC vial can be used in up to four patients.

We are only too aware of our small sample, and recommend that more studies with larger number of patients, incorporating parametric statistical testing, are needed to further corroborate our findings.

We are confident that in the future cyanoacrylate can be tried for rectus resections in addition to recessions. Its use should also be considered for re-attaching the conjunctiva to make the strabismus surgery completely sutureless.

Our study, therefore, provides evidence that supports the successful use of cyanoacrylate as a suitable bio-tolerant alternative to sutures to re-attach rectus muscles during recessions in strabismus surgery.

## References

[1] Remy C, Aracil P (1984). History of strabismus surgery. J Fr Ophthalmol.

[2] Forseth M, O’Grady K, Toriumi DM (1992). The current status of cyanoacrylate and fibrin tissue adhesives. J Long Term Eff Med Implants.

[3] Panda A, Kumar S, Kumar A, Bansal R, Bhartiya S (2009). Fibrin glue in ophthalmology. Indian J Ophthalmol.

[4] Marticorena J, Rodríguez-Ares MT, Touriño R, Mera P, Valladares MJ, Martinez-de-la-Casa JM (2006). Pterygium surgery: Conjunctival autograft using a fibrin adhesive. Cornea.

[5] Jiang J, Yang Y, Zhang M, Fu X, Bao X, Yao K (2008). Comparison of fibrin sealant and sutures for conjunctival autograft fixation in pterygium surgery: One-year follow-up. Ophthalmologica.

[6] Dadeya S, Ms K (2001). Strabismus surgery: Fibrin glue versus vicryl for conjunctival closure. Acta Ophthalmol Scand.

[7] Mohan K, Malhi RK, Sharma A, Kumar S (2003). Fibrin glue for conjunctival closure in strabismus surgery. J Pediatr Ophthalmol Strabismus.

[8] Erbagci I, Bekir N (2007). Sutureless closure of the conjunctiva with a commercial fibrin sealant in extraocular muscle surgery for strabismus. Strabismus.

[9] Marone P, Monzillo V, Segu C, Antoniazzi E (1999). Antibiotic-impregnated fibrin glue in ocular surgery: *In vitro* antibacterial activity. Ophthalmologica.

[10] Smiddy WE, Glaser BM, Green WR, Connor TB, Roberts AB, Lucas R (1989). Transforming growth factor beta.A biologic chorioretinal glue. Arch Ophthalmol.

[11] Banitt M, Malta JB, Soong HK, Musch DC, Mian SI (2009). Wound integrity of clear corneal incisions closed with fibrin and N-butyl-2-cyanoacrylate adhesives. Curr Eye Res.

[12] Beam JW (2008). Tissue adhesives for simple traumatic lacerations. J Athl Train.

[13] Lim LT, Bhatt PR, Ramaesh K (2008). Harvesting keratolimbal allografts from corneoscleral buttons: A novel application of cyanoacrylate adhesive. Br J Ophthalmol.

[14] Ricci B, Ricci F, Bianchi PE (2000). Octyl 2-cyanoacrylate in sutureless surgery of extraocular muscles: An experimental study in the rabbit model. Graefes Arch Clin Exp Ophthalmol.

[15] Chen WL, Lin CT, Hsieh CY, Tu IH, Chen WY, Hu FR (2007). Comparison of the bacteriostatic effects, corneal cytotoxicity, and the ability to seal corneal incisions among three different tissue adhesives. Cornea.

[16] Romero IL, Malta JB, Silva CB, Mimica LM, Soong KH, Hida RY (2009). Antibacterial properties of cyanoacrylate tissue adhesive: Does the polymerization reaction play a role?. Indian J Ophthalmol.

[17] Trott AT (1997). Cyanoacrylate tissue adhesive: An advance in wound care. JAMA.

[18] Dunlap EA, Dunn H, Rossomondo R (1969). Adhesive for sutureless muscle surgery. Arch Ophthalmol.

[19] Mulet ME, Alió JL, Mahiques MM, Mahiques MM, Martín JM (2006). Adal-1 bioadhesive for sutureless recession muscle surgery: A clinical trial. Br J Ophthalmol.

[20] Munton CG (1971). Tissue adhesive in ocular surgery: A prospective study. Exp Eye Res.

[21] Tonelli E, de Almeida HC, Bambirra EA (2004). Tissue adhesives for a sutureless fadenoperation: An experimental study in a rabbit model. Invest Ophthalmol Vis Sci.

